# Solution‐Processed Organic Photovoltaics: Fabrication Advances and Challenges

**DOI:** 10.1002/advs.202523048

**Published:** 2026-02-03

**Authors:** Kerui Liu, Chenyujie Zhu, Yuanyuan Jiang, Feng Liu, Xiaozhang Zhu

**Affiliations:** ^1^ Research Center for Future Organic Optoelectronics Global Institute of Future Technology (GIFT) Shanghai Jiao Tong University Shanghai China; ^2^ Institute of Chemistry Chinese Academy of Sciences Beijing P. R. China; ^3^ Beijing National Laboratory for Molecular Sciences CAS Key Laboratory of Organic Solids Institute of Chemistry Chinese Academy of Sciences Beijing P. R. China; ^4^ School of Chemical Sciences University of Chinese Academy of Sciences Beijing P. R. China

**Keywords:** device fabrication, electrodes, large‐area devices, organic solar cells, photoactive layers, transporting layers

## Abstract

The growing demand for sustainable energy solutions has propelled organic solar cells (OSCs) into the spotlight as a promising alternative to traditional inorganic photovoltaics. Despite their advantages—such as lightweight, flexibility, and semitransparency—OSCs face significant challenges related to efficiency and stability. This review provides a comprehensive overview of OSC device fabrication, focusing on the critical roles of photoactive layers, transporting layers, and electrodes in influencing performance. We explore recent advancements in material processing techniques and scalable manufacturing methods, particularly for large‐area devices, while addressing key issues like morphology optimization and charge carrier dynamics. By synthesizing current research trends and identifying areas for future investigation, this review aims to inform and inspire ongoing efforts in the field. The unique contribution of this manuscript lies in its detailed analysis of fabrication processes and the potential for enhanced efficiency and application in sustainable energy technologies, offering valuable insights for researchers and industry stakeholders alike.

## Graphical abstract

This review highlights recent advances in organic photovoltaic (OPV) device fabrication, emphasizing how material selection, interface engineering, and scalable processing techniques shape device performance. It discusses progress in photoactive and charge transport layers, electrode optimization, and large‐area manufacturing, offering insights into strategies that enhance efficiency, stability, and practical applicability of OPVs.

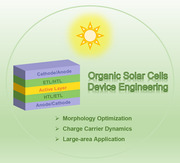

## 1 Introduction

The pursuit of sustainable energy sources has become increasingly critical in the face of escalating global energy demands and environmental challenges [1]. Among various renewable energy technologies, organic solar cells (OSCs) have emerged as a promising alternative to traditional inorganic solar cells. With their unique advantages, such as lightweight, flexibility, and low‐cost manufacturing processes, OSCs offer innovative solutions for diverse applications, including building‐integrated photovoltaics, portable electronic devices, and even wearable technology [2, 3, 4]. This review aims to provide a comprehensive overview of the device fabrication processes of OSCs, focusing on the processing of active layers, transport layers, electrodes, and the development of large‐area devices.

The fundamental operating principle of OSCs involves the absorption of sunlight by organic semiconductors, which leads to the generation of excitons—bound electron‐hole pairs [5]. The efficient separation and transport of these charge carriers to the respective electrodes are crucial for optimizing the overall power conversion efficiency (PCE) of the device. Unlike traditional solar cells that rely on rigid and heavy materials, OSCs utilize organic materials, which can be processed using low‐cost techniques such as solution processing and printing methods. This not only reduces production costs but also enables the fabrication of lightweight and flexible solar cells that can be integrated into various substrates, ranging from textiles to architectural surfaces. Despite the inherent advantages of OSCs, the technology is still in its developmental stages and faces several challenges. One of the primary hurdles is achieving high efficiency and stability comparable to that of inorganic solar cells. The performance of OSCs is highly dependent on the quality of the active layer, which typically comprises a blend of donor and acceptor materials. The optimization of active layer morphology is essential to enhance light absorption and facilitate effective charge separation and transport. Additionally, the design and fabrication of transport layers play a pivotal role in ensuring efficient electron and hole mobility, thus influencing the overall device performance. The processing of electrodes is equally critical, as it affects the charge extraction efficiency and overall energy conversion performance. The choice of electrode materials, their thickness, and the deposition methods used can significantly impact the device's performance.

Another significant area of research is the development of large‐area organic solar cells [6, 7]. While small‐scale devices have demonstrated promising efficiencies, scaling up fabrication processes to produce large‐area modules presents unique challenges. Achieving uniformity in material deposition, maintaining consistent active layer morphology, and ensuring the stability of large‐area devices are critical factors that require attention. Recent advancements in roll‐to‐roll processing and other scalable manufacturing techniques have shown potential in addressing these challenges, paving the way for practical applications of OSCs in commercial settings. Therefore, the device fabrication of OSCs encompasses a complex interplay of materials, processing techniques, and device architecture. This review aims to delve into the various aspects of OSC fabrication, focusing on the critical role of active layers, transport layers, and electrodes in determining the performance of these devices. Furthermore, it will address the emerging trends and future directions in the development of large‐area organic solar cells, highlighting the ongoing research efforts to enhance their efficiency, stability, and scalability. By providing a comprehensive overview of the current state of research in OSC device fabrication, this review seeks to inform and inspire future investigations in this rapidly evolving field, ultimately contributing to the advancement of sustainable energy technologies.

## 2 The Development of Photoactive Layers

### 2.1 The Development of Polymer Donors

**FIGURE 1:**
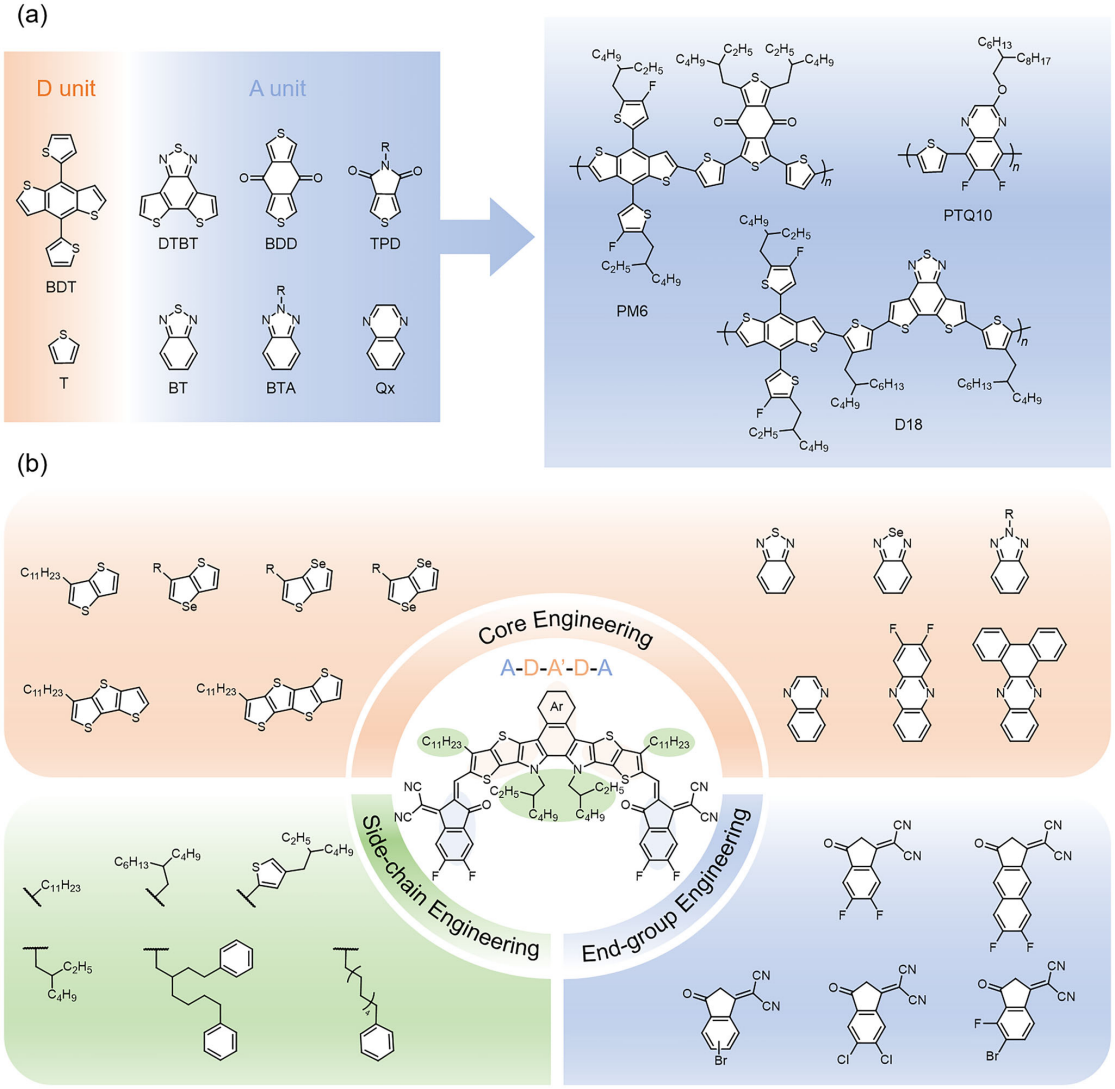
(a) The development of polymer donors. (b) The development of electron acceptors.

One of the primary challenges in developing optimal donor materials is the design and synthesis of conjugated polymers that exhibit excellent film‐forming properties, strong absorption, high hole mobility, and appropriate HOMO−LUMO energy levels [8]. Numerous donor‐acceptor (D‐A) conjugated polymers have been synthesized, significantly enhancing the PCE of OSCs. Key building blocks, such as benzodithiophene (BDT) [9], benzo[d][1,2,3]triazole (BTA) [10], benzothiadiazole (BT) [11], quinoxaline (Qx) [12], thieno[3,4‐c]pyrrole‐4,6‐dione (TPD) [13], thieno[3,4‐b]thiophene (TT) [14], and benzo[1,2‐c:4,5‐c′]dithiophene‐4,8‐dione (BDD) [15], have shown substantial benefits in developing high‐performance polymer donors (Figure 1a). Advances in molecular design have led to polymers like PM6 and D18, which have achieved PCEs exceeding 19% when paired with non‐fullerene acceptors (NFAs) [16, 17, 18]. Additionally, cost‐effective polymer donors such as PTQ10 and PTVT‐T demonstrate thickness insensitivity and high efficiency, which are promising for large‐area device fabrication [19, 20].

A deep understanding of molecular design, along with versatile polymer synthesis, allows for precise adjustments of conjugated polymers' intrinsic properties to meet specific application needs. For ideal polymer donors, creating complementary absorption spectra relative to designated electron acceptors is essential for maximizing solar photon utilization and achieving high *J*_SC_. Predictions indicate that PCEs above 19% could be attainable in single‐junction OSCs with onset absorption around 860 ± 60 nm [21]. Specialized applications impose stringent optical bandgap requirements: for instance, high‐performance tandem OSCs require bandgaps of approximately 1.70 eV and 1.20 eV for the front and rear cells, respectively [22]; an OSC intended for indoor use requires an active layer with an onset absorption wavelength below 700 nm [23]; an optimal semitransparent OSC should feature an active layer with visible transmission values reaching up to 50% (370–740 nm), while effectively capturing light with wavelengths shorter than 435 nm and longer than 670 nm [24]. Overall, controlling the bandgap of polymer donors is crucial for meeting the diverse requirements of OSC applications and advancing performance limits in future commercialization efforts.

### 2.2 The Development of Electron Acceptors

Electron acceptors are essential components of the active layer in OSCs. Fused‐ring electron acceptor (FREA) materials—primarily comprising fused aromatic diimide derivatives such as perylene diimides (PDIs) and naphthalene diimides (NDIs), fullerene acceptors, A‐D‐A/A‐DA'D‐A type NFAs—have significantly contributed to the advancement of OSCs [25].

NFAs have revolutionized OSC performance by offering greater flexibility in molecular design [26]. The structural diversity of each unit (Figure 1b) offers significant flexibility in regulating optoelectronic properties, blend morphology, and ultimately device performance. A common strategy for core design is to modify the conjugation length, which influences absorption, energy levels, and molecular stacking [27]. Another approach involves introducing heteroatoms into the existing core [28]. Due to their varying electronegativities, heteroatoms can effectively adjust the electron‐donating capability of the cores, thereby impacting the optoelectronic properties of the acceptors. Additionally, heteroatoms may provide extra sites for side chains, affecting solubility and molecular stacking. In 2019, our group designed and synthesized AQx‐type acceptors featuring a quinoidal‐enhancing Qx moiety, achieving a record‐certified PCE at the time of publication [29]. Recently, AQx‐type acceptors have garnered considerable attention due to their superior core functionalization compared to Y‐type acceptors with benzothiazole moieties [30, 31, 32]. Among them, AQx‐2F‐based binary devices achieved a superior PCE of 19.7% [31]. 83% of the initial PCE of the device can be retained after 500 h, presenting decent operational stability. To fine‐tune the electron‐accepting capabilities of terminal groups, an effective method is to attach electron‐donating or ‐withdrawing groups [33]. Furthermore, side‐chain engineering plays a crucial role in enhancing solubility and influencing molecular stacking in solid films, which is vital for developing high‐performance NFAs [34].

Except for the small‐molecular FREAs as mentioned above, nonfused ring acceptors with simplified synthesis and polymer acceptors with mechanical flexibility and thermal stability have gathered wide attention [35, 36]. The development of NFAs represents a paradigm shift in OSC technology, offering a path forward for higher efficiency, improved stability, and the potential for low‐cost, scalable production. The continuous innovation in NFA design is expected to remain a driving force in the advancement of OSC performance.

### 2.3 The Processing of Active Layers

#### 2.3.1 The Solvent

Solution‐processable organic semiconductors present promising opportunities for sustainable manufacturing [37]. The choice of solvent is critical in the fabrication of OSCs, as it directly affects the morphology of the photoactive layer, which in turn influences the overall efficiency of the device. As a general guideline, the solvent must effectively solubilize all components in the blend films at sufficient concentrations to form continuous films of the desired thickness and properly wet the surfaces of the intended substrates.

One of the key challenges in solvent selection is balancing the solubility of both the donor and acceptor materials to achieve an ideal nanoscale phase separation that promotes efficient exciton dissociation and charge transport. Moreover, solvent volatility plays a significant role in controlling the drying kinetics during active layer deposition. Highly volatile solvents may lead to rapid drying and inadequate molecular organization, resulting in poor phase separation and suboptimal morphology. On the other hand, low‐volatility solvents provide a longer drying time, which allows for better self‐assembly of the donor and acceptor materials into favorable morphologies. This is especially critical for achieving optimal crystallinity in high‐performance systems, where a balance between slow crystallization and sufficient mixing must be maintained for high charge mobility. Additionally, other solvent properties, including boiling point, surface tension, viscosity, and polarity, should be considered, as these factors significantly influence film drying kinetics and morphology [38, 39].

**FIGURE 2:**
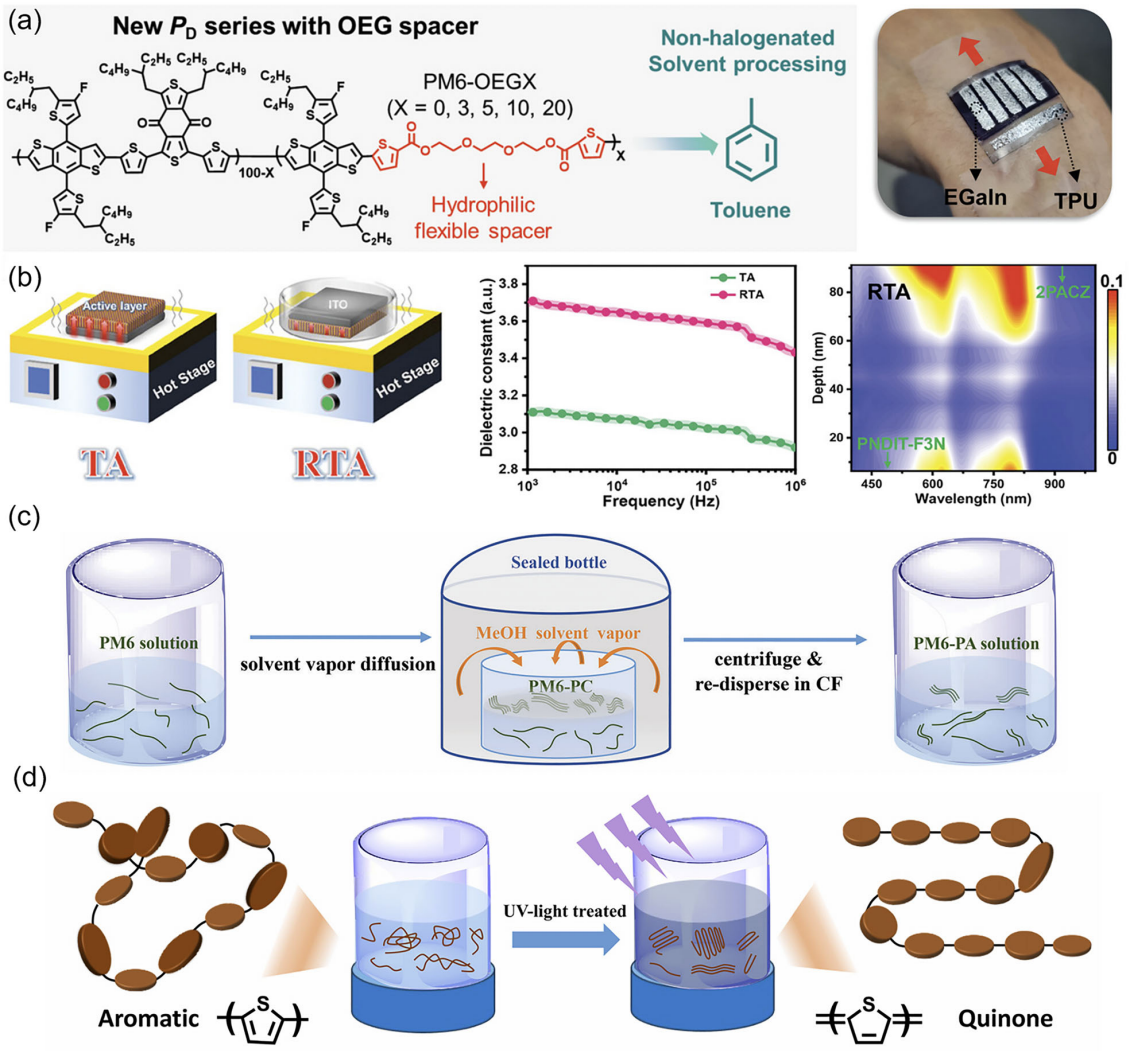
(a) The material design for the non‐halogenated‐solution process and flexible device fabrication. Reproduced with permission from Ref. [51]. Copyright 2022, Wiley‐VCH. (b) The RTA post‐treatment for improved dielectric constant and the vertical phase segregation. Reproduced with permission from Ref. [56]. Copyright 2024, Wiley‐VCH. (c) Schematic diagram of the preparation of PM6‐PA solution. Reproduced with permission from Ref. [61]. Copyright 2023, Wiley‐VCH. (d) Schematic diagram of light‐induced pre‐aggregation of polymer donors. Reproduced with permission from Ref. [62]. Copyright 2024, Royal Society of Chemistry.

Currently, most OSCs are prepared by halogenated solvents, such as chloroform, chlorobenzene, and 1,2‐dichlorobenzene. However, these solvents are highly toxic and not environmentally friendly, and their costs are generally higher than those of non‐halogenated alternatives. Therefore, there has been growing interest in developing non‐chlorinated, environmentally friendly solvents as alternatives to chlorobenzene and other toxic options. Solvents such as o‐xylene, tetrahydrofuran, and even greener alternatives like 2‐methyl tetrahydrofuran and cyclopentyl methyl ether have been investigated for their ability to form appropriate active layer morphologies without compromising device efficiency [40, 41, 42]. Although these solvents typically show lower solubility for high‐performance donor and acceptor materials, advances in material design and solvent engineering have made it possible to achieve comparable efficiencies to those obtained using chlorinated solvents. Several strategies have been developed to modify the molecular structure of photoactive materials and reduce molecular aggregation for enhanced solubility in green solvents [43]. These strategies include increasing alkyl chain length [44, 45], introducing bulky structures or heteroatoms into the side chain [46, 47], twisting the backbone structure [48, 49], and employing ternary polymerization [50, 51]. Kim et al. developed a series of polymer donors (PM6‐OEGX) by introducing a hydrophilic oligo(ethylene glycol) flexible spacer (OEG)‐FS into the backbone of PM6 (Figure 2a) [51]. The hydrophilic flexible spacer increases the molecular flexibility and enables non‐halogenated solvent processing to realize a high PCE of 17.74%. However, excessive modification can adversely affect molecular packing and the electrical properties of photoactive materials, resulting in poorer morphology and reduced photovoltaic performance. Therefore, the molecular structure of photoactive materials must be carefully optimized to enable high PCE in green‐solvent‐processed OSCs.

The choice of solvent must be carefully tailored to the specific D‐A system in use, with considerations for solubility, drying dynamics, and the use of additives. As the field moves toward more sustainable and scalable fabrication methods, the development of non‐toxic solvents will be critical in pushing OSC technology toward commercial viability while maintaining high performance.

#### 2.3.2 The Additive

Additives are essential in the fabrication of OSCs as they provide a powerful means to control the morphology, phase separation, and crystallinity of the photoactive layer [52]. The delicate balance between the donor and acceptor phases within the bulk heterojunction (BHJ) is crucial for optimizing exciton dissociation, charge transport, and minimizing recombination losses. Additives, when introduced in small amounts, play a significant role in fine‐tuning these morphological parameters, leading to enhanced PCE. In the case of NFAs, which often exhibit strong self‐aggregation tendencies, additives are even more critical.

Additives can be broadly classified into solvent additives and solid additives. The common solvent additives contain 1,8‐diiodooctane (DIO), 1‐chloronaphthalene (CN), diphenyl ether (DPE), and o‐dichlorobenzene (o‐DCB). These high‐boiling‐point solvents can slow the crystallization process during film formation. Zhang et al. used DIO to help achieve a more favorable nanoscale phase separation in PBDB‐T‐2F:ITIC active layers, thereby facilitating more efficient charge transport pathways [53]. Beyond DIO, other additives have also been explored for their specific effects on different donor‐acceptor systems. Lu et al. found that CN can induce highly ordered packing of acceptor in PM6:Y6 blend films to achieve a favorable crystalline morphology [54]. The improved long‐range ordering can be attributed to the π–π interactions between naphthalene derivatives and terminals of Y6. The choice of solvent additive is often tailored to the specific donor‐acceptor combination, as the additive's solubility parameters must align with the solubility and crystallization kinetics of the active layer components. Moreover, the concentration of the solvent additive is critical; while low concentrations generally improve morphology, excessive amounts can disrupt the molecular ordering and result in inferior device performance. The influence of additives is not limited to morphology control alone; they can also impact the vertical distribution of the donor and acceptor materials within the active layer. This vertical stratification, where the donor material is more concentrated near the anode and the acceptor near the cathode, can enhance charge extraction efficiency and reduce recombination losses.

Recently, the development of solid additives has provided unique advantages in morphology control, post‐treatment ease, and device stability, leading to their rapid adoption as a universal method for optimizing performance in OSCs [55]. Solid additives in OSCs are generally classified into two categories: volatile and nonvolatile, based on their retention in the active layers. Volatile additives can be completely removed from the active layers through thermal annealing or alcohol washing, while nonvolatile additives remain due to their high boiling points or strong intermolecular forces. Regardless of their retention, both additives enhance the morphology of the active layer and boost OSC stability. The operational mechanisms of solid additives are crucial for device optimization. For volatile additives, thermal annealing primarily facilitates their removal, which increases the free volume within the donor and/or acceptor layers. This additional free volume fosters nanoscale phase separation and improved molecular organization, essential for enhancing charge transport and light absorption properties. Moreover, the performance of the device is significantly influenced by the interactions between solid additives and the donor or acceptor molecules. These interactions—primarily through hydrogen bonding, halogen bonding, and π−π interactions—play a key role in promoting the aggregation of donor or acceptor molecules, facilitating π−π stacking, and improving the formation of bicontinuous interpenetrating networks and vertical phase separation. Collectively, these factors contribute to the enhanced performance and stability of OSCs.

Additives play a critical role in enhancing the efficiency of OSCs by precisely controlling the morphology, phase separation, and crystallinity of the active layer. Their ability to fine‐tune these parameters at the nanoscale is essential for optimizing charge transport and minimizing recombination losses. As research continues to explore new additives and environmentally friendly alternatives, their role in advancing OSC performance and scalability remains indispensable.

#### 2.3.3 Thermal/Solvent Annealing

Post‐processing techniques, such as thermal annealing and solvent annealing, are widely employed in the fabrication of OSCs to enhance the molecular ordering, phase separation, and overall morphology of the active layer, which are critical for improving device performance [56, 57, 58]. Thermal annealing indicates heating the active layer after deposition to promote the crystallization of both the donor and acceptor materials, facilitating the formation of optimized pathways for charge transport. In particular, thermal annealing helps achieve a more favorable bulk BHJ morphology by inducing phase separation between the donor and acceptor, thereby improving exciton dissociation and charge extraction efficiency. Zhang et al. applied a reverse thermal annealing (RTA) technique to enhance the dielectric constant of the active layer (Figure 2b) [56]. This method enables a favorable nano‐scale phase aggregation and molecular stacking orientation and can reduce the decline in *V*_OC_ of the conventional TA method. The PCE of the PM6:L8‐BO‐X device increases from 18.98% of the TA device to 19.91% (certified 19.42%) of the RTA device.

The annealing temperature and duration are key factors that influence the final morphology of the active layer. For example, annealing at moderate temperatures (80–150°C) for optimal time periods can enhance the crystallization of low‐bandgap polymer donors such as PTB7 and PBDB‐T while preventing excessive phase separation that could lead to isolated donor or acceptor domains. However, excessive annealing temperatures or prolonged annealing times can negatively affect device performance by promoting over‐crystallization or morphological instability. Ye et al. could precisely manipulate the crystalline order in the active layer and achieve a remarkable 19‐fold efficiency increase by simply shortening the annealing time [57]. The P3HT:ZY‐4Cl blend film by thermal annealing for 30 s gave rise to a superior PCE of 10.7%.

In addition to thermal annealing, solvent annealing has emerged as a powerful technique to further optimize the morphology of OSC active layers. In solvent annealing, the active layer is exposed to solvent vapors that selectively swell one or more components of the donor‐acceptor blend, allowing for reorganization at the molecular level. This can effectively enhance molecular ordering and vertical stratification in the active layer, resulting in better electron and hole transport and reduced recombination losses. Wang et al. found that solvent annealing could be an effective method to enable increased coherence length value, higher percentage of crystalline regions, and reduced bimolecular recombination in the PTB7‐Th:PNDI‐T10 solar cells, leading to a remarkable increase in FF from 0.54 to 0.71 [58]. Solvent annealing can well control the evaporation rate of processed solvent, providing a suitable time for molecular assembly to form well‐organized nanostructures, which is promising for improved FF and *J*_SC_.

Both thermal and solvent annealing play vital roles in optimizing the morphology and molecular ordering of the active layer in OSCs. These post‐processing techniques are essential for achieving higher charge mobility, better phase purity, and ultimately, improved PCE in OSC devices. As the field progresses, these methods continue to be refined and adapted to the evolving landscape of high‐performance OSC materials and scalable production processes.

#### 2.3.4 Some Special Processing Methods

To provide a concise yet comprehensive introduction to the special processing methods employed in the fabrication of the photoactive layer for OSCs, it is essential to recognize the pivotal role these techniques play in enhancing morphology and performance. Traditional methods, such as single solvent selection and thermal annealing, set a foundational framework; however, recent advancements have led to the exploration of innovative strategies that delve deeper into material properties. For instance, the use of binary solvent systems allows for fine‐tuning of solubility parameters, thereby promoting better phase separation and crystallinity in the active layer. Liu et al. selected a binary solvent system (chloroform and o‐xylene) that exhibits different solubility for donor D18 and varied evaporation properties [59]. Upon the evaporation of chloroform, fibril structures are formed by D18, while acceptor molecules (2BTh‐2F‐C2) are still dissolved. Following the evaporation of o‐xylene, a rapid fibril network emerges, effectively segregating 2BTh‐2F‐C2 into a pure acceptor fibril phase within the remaining solvent. Utilizing the solvent mixture technique can mitigate excessive intermolecular mixing or phase separation, facilitating an optimal and clearly defined interpenetrating network morphology. Through this approach, D18:2BTh‐2F‐C2‐based solar cells achieved a PCE of 19.02% for small‐area devices and 17.28% for 1 cm^2^ units.

Before film deposition, several methods are available to handle the active layer solution in advance. Kim et al. found that simple filtration of the material solution can remove large aggregates from the solution without destroying the nanoscale preaggregation, facilitating enhanced acceptor ordering in the active layer and improving device operational stability [60]. Wang et al. also reported two methods to process the spin‐coated solution. One is preparing cylindrical PM6 polycrystals by a vapor diffusion method [61]. The PM6 polycrystals are redissolved in chloroform to create PM6 pre‐aggregates (PM6‐PA), which are then incorporated into traditional PM6:L8‐BO blend solutions (Figure 2c). This approach has been shown to extend the molecular organization process and improve the aggregation of both PM6 and L8‐BO components. Consequently, when using 10% PM6‐PA, the PCE of PM6:L8‐BO solar cell devices increased from 18.0% to 19.3% and from 16.2% to 17.2% for photoactive layers measuring 100 nm and 300 nm thick, respectively. The other is by irradiating polymer solutions with 365 nm UV light to enhance the intermolecular ordering of donors (Figure 2d) [62]. Raman spectroscopy and Fourier transform infrared spectroscopy demonstrate that this light irradiation disrupts the aromatic conformation of the polymers and induces the formation of a rigid quinone structure, which can improve the planarity of the polymers, leading to the formation of compact aggregates with enhanced π−π stacking in both pristine and blend thin films with the acceptor L8‐BO. Consequently, devices based on PM6:L8‐BO and D18:L8‐BO exhibited improved carrier transport and reduced recombination, resulting in PCE increases from 18.8% to 19.7% and 18.9% to 19.6%, respectively. Concerning some high‐boiling solvent‐processed devices, An et al. proposed a hot‐casting strategy for enhanced photovoltaic performance [63]. In the active layer spin‐coated by a hot solution, the donor and acceptor present the fast and synchronous molecular organization, facilitating favorable vertical phase separation and improved charge dynamics. The hot‐casting devices based on PM6:BTP‐eC9 in ortho‐xylene achieved a remarkable FF of 80.31% and a PCE of 18.52%.

These advanced processing methods improve the structural and electronic properties of the active layers and offer insights into the interplay between processing conditions and material performance, setting the stage for the ultimate development of high‐efficiency OSCs. Nevertheless, these approaches may exhibit certain limitations in terms of scalability and reproducibility, particularly as evidenced by the performance gap between small‐area and large‐area devices, which necessitates a critical examination of fluid dynamics and drying kinetics.

The continuous advancement of photoactive layers—spanning polymer donors, non‐fullerene acceptors, and various processing methods—has been fundamentally driven by the pursuit of optimized bulk‐heterojunction morphology. A well‐defined nanoscale phase separation with balanced domain purity and interconnectivity is critical not only for efficient exciton dissociation but also for enabling unimpeded charge transport and collection. Therefore, establishing quantitative morphology–performance relationships and elucidating the underlying charge‐transport physics have become central themes in modern OSC research. For instance, Ade et al. established a critical thermodynamic link between blend miscibility and device performance in OSCs [64]. By combining phase‐diagram mapping via secondary‐ion mass spectrometry (SIMS) with device analysis, the authors quantitatively determine the amorphous–amorphous interaction parameter *χ*_aa_(T) for a model polymer:fullerene system. They demonstrate a characteristic “constant‑kink‑saturation” relationship between *χ*_aa_ and FF: a sufficiently large *χ*_aa_ drives strong phase separation and high domain purity, leading to suppressed charge recombination and maximized FF. This correlation is further validated across multiple donor:acceptor systems, providing a generalizable criterion for predicting morphologically limited performance. The work highlights that rapid screening of *χ*_aa_ (e.g., via DSC) can guide material selection and reduce trial‑and‑error in device optimization, offering a thermodynamics‑based roadmap for achieving high‑efficiency organic blends. Beyond thermodynamics, the charge transport behavior within the blend is equally decisive; the field‐dependent mobility, energetic disorder, and interfacial trap states collectively determine the extraction efficiency and final device performance. Recent works combining drift‐diffusion simulations, temperature‐dependent mobility measurements, and energy‐loss analysis have provided deeper insights into how nanoscale morphology dictates charge‐carrier dynamics and voltage losses [65, 66, 67]. Liu et al. demonstrated a refined double‐fibril network morphology in the PM6:D18:L8‐BO ternary system, which significantly promotes charge transport [68]. In this morphology, densely interpenetrated donor and acceptor fibrils form continuous, high‐speed pathways for holes and electrons. Critically, the characteristic size of the intermixed phase within the fibril network is optimized to match the exciton diffusion length, ensuring efficient exciton dissociation and subsequent carrier diffusion into the crystalline transport channels. This morphology‐physics synergy leads to balanced carrier transport, suppressed recombination, and a remarkable fill factor approaching 82%, contributing to a certified PCE of over 19%. Moving forward, coupling such theoretical and experimental tools with high‐throughput material screening will be essential to rationally design photoactive systems that simultaneously achieve optimal morphology, efficient charge transport, and high operational stability.

## 3 The Development of Transporting Layers

### 3.1 The Development of Hole Transporting Layers

**FIGURE 3:**
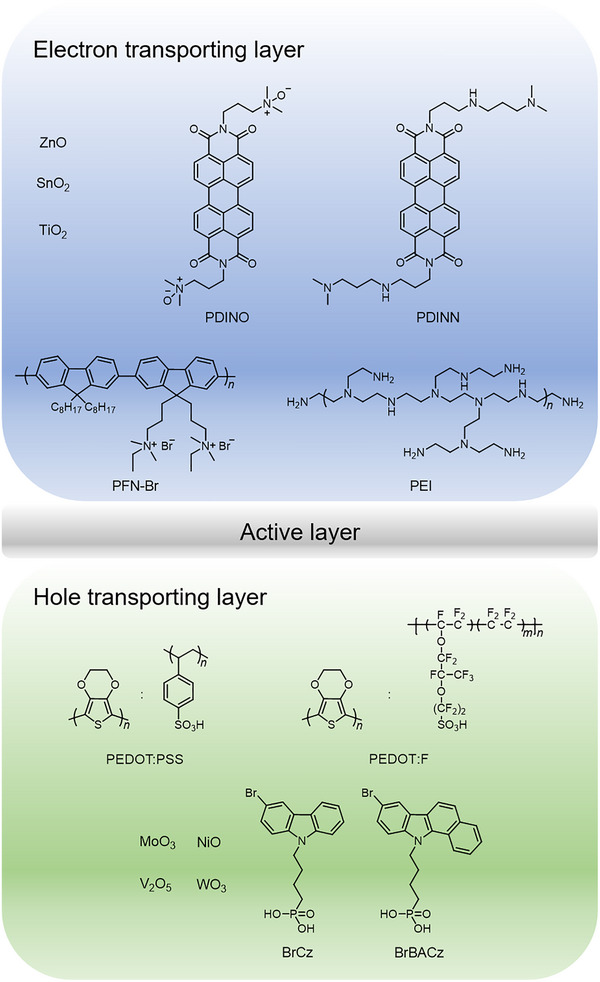
The common transporting materials in OSCs.

By inserting proper transporting layer materials (Figure 3) and advancing interfacial engineering between the active layer and the electrodes, the work functions of both electrodes can be precisely adjusted, and the contact interface between the active layer and the electrodes can be optimized [69]. This results in a beneficial cascade energy alignment that significantly improves charge extraction efficiency and overall photovoltaic performance. Hole transporting layers (HTLs) are integral to the performance of OSCs, serving to efficiently transport positive charges (holes) generated by light absorption in the active layer. Till now, poly(3,4‐ethylenedioxythiophene):polystyrene sulfonate (PEDOT:PSS) remains the predominant choice for HTLs in traditional OSCs, due to the advantages of suitable WF, water solubility, good mechanical properties, high optical transparency, and wettability for BHJ solutions [70]. However, the limitations of PEDOT:PSS, including its hygroscopic nature and susceptibility to photo‐oxidation, prompted researchers to explore modified PEDOT:PSS (solvent‐modified PEDOT:PSS, metal oxide‐modified PEDOT:PSS, surfactant‐modified PEDOT:PSS, and PEDOT‐based derivatives) that offer improved stability and performance [71, 72, 73, 74].

Alongside modifying the properties of PEDOT:PSS, there has been a growing interest in developing alternative organic and inorganic HTLs. For instance, (1) conjugated polymer‐based HTLs, which include various conjugated polymers and polyelectrolytes [75]; (2) transition metal oxide‐based HTLs, such as MoO_3_, NiO, V_2_O_5_, and WO_3_, that are characterized by high work functions and excellent electrical conductivity, typically deposited onto ITO substrates via thermal deposition methods [76]; (3) Polyoxometalate‐based HTLs, which represent a broad family of water‐soluble inorganic clusters composed of transition metal‐oxygen anion complexes with a relatively rigid cage‐like structure [77]. Notably, Zhou et al. introduced an alcohol‐dispersed conducting polymer complex, PEDOT:F, which uses perfluorinated sulfonic acid ionomers as counterions [78]. This formulation overcomes the limitations of traditional aqueous PEDOT:PSS, such as strong acidity and poor wetting properties. The alcohol‐dispersed PEDOT:F exhibits better wetting and lower acidity, enabling fully printable organic photovoltaics with improved efficiency and stability. Additionally, (1) graphene oxide (GO), an oxidized form of graphene, is functionalized with various oxygen groups like epoxy and hydroxyl, and can be readily synthesized through the chemical oxidation of naturally occurring graphite [79]. Compared to PEDOT:PSS, GO serves as a more transparent interfacial material, offering benefits such as solution processability, a unique 2D architecture, and an adaptable electronic structure. (2) MXenes, a novel class of 2D materials made from transition metal carbides or nitrides, have garnered significant interest due to their high conductivity, flexibility, and hydrophilicity [80]. The work function of MXenes is highly influenced by their surface functional groups, allowing for regulation to meet the needs of electronic device applications. (3) Self‐assembled monolayers (SAMs) featuring sulfonic, phosphoric, and carboxylic acid groups emerge as promising candidates for interfacial modification [81]. Ge et al. designed and synthesized two SAMs containing phosphonic groups, named BrCz and BrBACz. Compared with BrCz, BrBACz exhibits enhanced dipole moment, reduced surface energy, and deeper work function. The corresponding devices using BrBACz show improved photon harvesting of the active layer, minimized voltage losses, and optimized interface charge extraction/transport [82].

Ongoing research is focused on optimizing processing techniques, including solution processing and vapor deposition, to achieve uniform and defect‐free films. This multidisciplinary approach to HTL development is crucial for enhancing the overall efficiency and longevity of OSCs, ultimately contributing to their competitiveness in the renewable energy landscape.

### 3.2 The Development of Electron Transporting Layers

Qualified electron transporting layer (ETL) materials must exhibit several essential characteristics: they should lower the cathode's work function to align with the LUMO level of the acceptors, possess efficient electron transport and effective hole‐blocking capabilities, and demonstrate suitable compatibility with both the active layer and the cathode [83]. These properties are crucial for enhancing the efficiency of OSCs by promoting effective electron extraction and reducing recombination losses. The development of ETL materials has continuously progressed, encompassing metal oxides such as ZnO, SnO_2_, and TiO_2_; organic small molecules, particularly derivatives of perylene diimide (PDI); polymers like PFN‐Br and PEI; as well as composites and hybrids of these various materials.

Currently, the development of metal oxide nanoparticles (NPs) with improved processability and reduced sensitivity to fabrication conditions is ongoing and widely applied. Additionally, inverted devices utilizing metal oxide ETLs exhibit excellent long‐term stability [84]. However, metal oxides like ZnO and SnO_2_ tend to have significant oxygen vacancy defects within their crystal structures, leading to non‐radiative recombination. This phenomenon impedes effective charge transport and collection, adversely affecting the thickness sensitivity and stability of devices. To address these issues, organic‐modified metal oxides and organic‐protected metal oxides have been developed for ETL applications. In conventional devices, perylene diimide (PDI) and naphthalene diimide (NDI)‐based ETLs are extensively researched due to their energy level alignment and good Ohmic contact with the cathode [85]. Among these, PDINO is frequently employed with aluminum cathodes; however, aluminum's stability in air is limited. Consequently, Li et al. synthesized a novel PDI‐based material, PDINN, which features a functionalized aliphatic amine group [86]. This material not only lowers the work function of silver and copper electrodes but also forms hydrogen bonds with the active layers, enhancing interfacial contact. Furthermore, PFN‐Br has garnered attention due to its solubility in alcohol, suitable dipole moment, and capability to reduce the work function of silver and other air‐stabilized metals in conventional devices, as well as ITO in inverted devices [87]. Notably, Wen et al. demonstrated that electrons can be efficiently transferred from the amine groups of amine‐containing materials like PDINO and PFN‐Br to NFAs, leading to undesirable structural changes in both materials under illumination [88]. To mitigate this, they proposed a high‐stability, phenol‐functionalized perylene bisimide (PBI) as an amine‐free ETL alternative. Additionally, hybrid systems have been explored, with Liu et al. combining transition metal complexes with carbon‐based materials, resulting in a series of metal‐nanographene complexes (HBC‐H, HBC‐P, HBC‐S) featuring varying π‐conjugate lengths [89].

Solution‐processing techniques, such as sol‐gel and spray pyrolysis, have facilitated the deposition of these materials, allowing for large‐area applications. Ongoing research continues to focus on optimizing the interface between the ETL and the active layer to ensure effective charge separation and transport. Collectively, these developments are crucial for advancing the performance and commercial viability of OSCs in the renewable energy landscape.

Notably, the selection of the interfacial layer not only directly affects the charge extraction efficiency of the device but is also a critical factor determining its long‐term operational stability. Taking the most commonly used hole transport material PEDOT:PSS as an example, while it offers advantages such as high conductivity and favorable energy level alignment, its inherent hydrophilicity and acidity can lead to significant performance degradation under humid conditions. This instability primarily stems from the hygroscopic nature of PEDOT:PSS and its corrosive effect on underlying electrodes, such as ITO. In contrast, inorganic interfacial materials (e.g., MoO_3_, V_2_O_5_, NiO_x_) demonstrate considerable potential for enhancing device environmental stability due to their dense thin‐film morphology and excellent chemical stability. However, inorganic materials often require vacuum evaporation for deposition, which involves higher processing costs. Additionally, their film morphology may not form as perfect an interfacial contact as spin‐coated organic layers, sometimes resulting in a slight compromise in absolute efficiency. Therefore, the core of interfacial engineering lies in balancing efficiency and stability.

### 3.3 The Processing of Transporting Layers

#### 3.3.1 The Solution Processing of Organic Transporting Layers

Solution processing of organic transport layers in OSCs offers significant advantages, particularly in terms of cost‐effectiveness and scalability, making it a favored method in the fabrication of these devices. A Sequential deposition strategy of electrodes, interlayer layers, and active layers is generally applied in the device processing. Spin‐coating is the most commonly employed method for the solution processing of transporting layers, where a small amount of the organic solution is deposited on the substrate, followed by rapid spinning to achieve a uniform thin film. This technique is highly controllable, allowing for precise adjustment of parameters such as processing solvent, solution concentration, and spin speed to achieve the desired film thickness (10–50 nm) and morphology. The majority of organic transporting materials are processed using environmentally friendly solvents, primarily polar organic solvents such as ethanol, DMF, and NMP. Zhu et al. proposed a solvent‐induced anti‐aggregation (SIAA) strategy, addressing the inherent aggregation issue of the ETL layer [90]. By the use of mixed solvents of ethanol and trifluoroethanol, the solubility, aggregation, and electronic properties of the ETL layer were finely tuned, leading to enhanced film quality and electron‐transport capability. The corresponding devices based on PM6:L8‐BO achieved improved stability and a champion PCE of 19.0% with an impressive FF of 80.6%. A 1 cm^2^ device demonstrating an impressive PCE of 16.6% has been fabricated using doctor‐blading techniques with SIAA‐treated PDINN ink. Furthermore, post‐treatments, such as thermal annealing and UV‐ozone, can enhance the film‐forming ability, the film morphology, and the work function, leading to improved conductivity.

**FIGURE 4:**
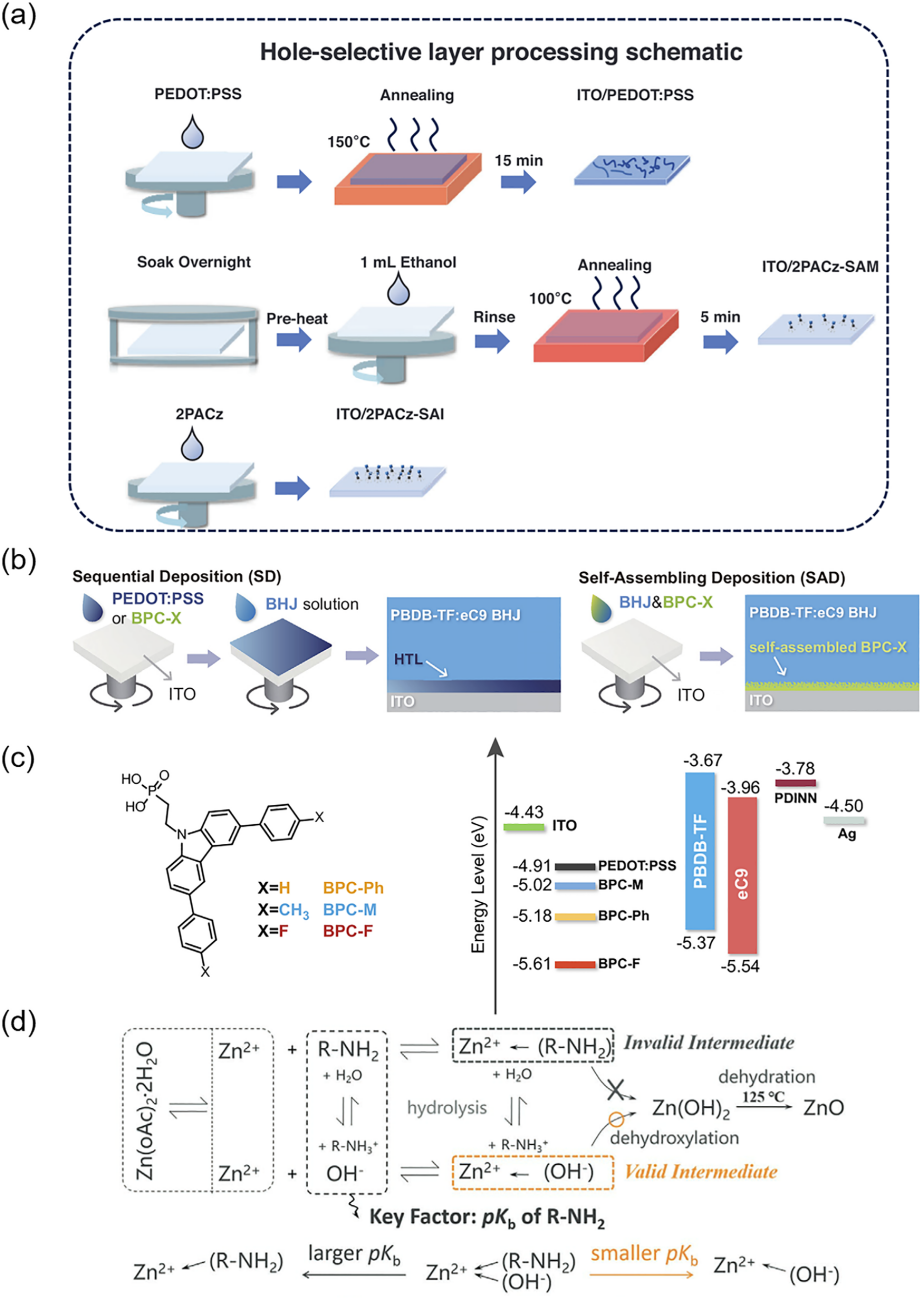
(a) The processing protocols of three types of hole‐transporting layers: PEDOT:PSS, 2PACz‐SAM, and 2PACz‐SAI. Reproduced with permission from Ref. [91]. Copyright 2024, Wiley‐VCH. (b) Schematics of SD and SAD processing. (c) Chemical structures of the SAMs and the energy diagram showing the altered work function. Reproduced with permission from Ref. [92]. Copyright 2024, Elsevier. (d) The generation of wurtzite ZnO via sol–gel technology. Reproduced with permission from Ref. [104]. Copyright 2022, Wiley‐VCH.

In particular, Chen et al. reported the creation of a self‐assembled interlayer (SAI) through a straightforward one‐step spin‐coating process using the self‐assembled molecule 2‐(9H‐carbazol‐9‐yl) (2PACz), without the need for any additional treatment (Figure 4a) [91]. The SAI forms a thin layer measuring 2–6 nm, consisting of multiple layers of self‐assembled molecules that completely cover the ITO substrate via both covalent bonding and van der Waals forces. When compared to PEDOT:PSS, the 2PACz‐SAI significantly enhances surface and interface optoelectronic properties, including optical field distribution, light absorption, and carrier dynamics, resulting in an improved efficiency from 19.19% to 20.17% (certified at 19.79%) in the PBDB‐TF:L8‐BO:BTP‐eC9 system. Furthermore, the incorporation of ITO/2PACz‐SAI proves particularly beneficial for enhancing light absorption selectivity in semi‐transparent photovoltaics (ST‐OSC), achieving a record light utilization efficiency of 5.34%. The bifacial ST‐OSC demonstrates comparable performance under both indoor (500 lux) and outdoor (1 sun) conditions, indicating its potential for practical applications. Moreover, Hou et al. synthesized a series of self‐assembled molecules, achieving an excellent PCE of 19.3% in binary OSC by a self‐assembling deposition (SAD) strategy (Figure 4b) [92]. This approach utilizes self‐assembled charge‐transport materials mixed in the solution alongside active layer materials, allowing for the concurrent deposition of these two layers, which can simplify OSC fabrication. Interfacial energy level alignment is critical for achieving efficient charge extraction. Interface modification materials such as SAMs enable precise tuning of the electrode work function through their intrinsic dipole moments (Figure 4c), thereby establishing energy level gradients or Ohmic contacts between the active layer and the electrode, which significantly reduces the energy barrier for charge extraction. Consequently, the strategic pairing of electrodes with suitably matched transport layers is essential for maximizing charge collection efficiency and overall device performance.

Compared to vacuum deposition, solution processing of organic transport layers is a vital aspect of OSC fabrication, offering advantages in cost and scalability but generally exhibiting greater defect density. The ongoing research into optimizing processing parameters and exploring new materials will continue to drive improvements in device performance and pave the way for more widespread adoption of OSC technology in the renewable energy sector.

#### 3.3.2 The Solution Processing of Inorganic Transporting Layers

Solution processing of inorganic transport layers in OSCs represents a distinct approach compared to organic materials, primarily due to the unique properties and requirements of inorganic compounds, such as metal oxides. These materials, including titanium dioxide (TiO_2_) and zinc oxide (ZnO), offer several advantages, including high electron mobility and excellent light transmittance, making them ideal candidates for use as ETLs in inverted devices.

The solution processing of these inorganic layers often employs methods such as nanoparticle dispersion [93] or sol‐gel processing [94], which facilitates the formation of thin films with tailored properties. However, nanoparticles often aggregate into larger clusters during preparation and storage, resulting in considerable reproducibility challenges [95]. The sol‐gel method offers more control in synthesizing metal oxides, yet it typically requires high‐temperature post‐thermal annealing above 200°C to convert precursors into the desired metal oxides. This process is not only energy‐intensive but also incompatible with flexible substrates [96]. This process allows for the formation of highly uniform and porous films that can be adjusted for thickness and morphology by controlling parameters such as precursor concentration, temperature, and pH. However, metal oxides can exhibit numerous surface defects and significant photocatalytic activity, which may lead to poor interfacial contact and compatibility with the photoactive layer, as well as accelerate degradation processes. For example, the unintentional introduction of impurities such as oxygen vacancies or hydrogen ions can lead to n‐type doping in ZnO. This doping effect may induce undesirable space‐charge formation near the cathode, which in turn contributes to significant efficiency losses via interfacial recombination. Passivation of the metal oxide layer represents a promising strategy to mitigate these challenges [97, 98, 99, 100, 101, 102, 103]. Hou et al. introduced a novel method for creating printable ZnO to be used as an ETL in flexible OSCs (Figure 4d) [104]. They utilized three different amines—ethanolamine (EA), n‐propylamine (PA), and triethylamine (TEA)—as Lewis bases in the precursors to synthesize three types of ZnO (EA‐, PA‐, and TEA‐ZnO) through pKb‐dependent sol‐gel reactions. The sol‐gel precursor containing PA was particularly well‐suited for blade coating, as it helped prevent the formation of bumps and coffee ring effects. Additionally, the stronger Lewis base resulted in a higher growth draw ratio, an increased proportion of valid intermediates in the precursor, and a reduced proportion of polar facets in the final ZnO. Consequently, a lower thermal annealing temperature of 130°C was sufficient to convert the PA‐based gel into ZnO, positioning PA‐ZnO as an ideal candidate for use as an ETL in flexible OSCs.

Therefore, it is essential to prepare metal oxide‐based transporting layers from appropriate precursors under milder conditions while effectively passivating defects to enhance their future applications. By optimizing processing parameters and exploring innovative material combinations, researchers can significantly enhance the performance and stability of OSCs, paving the way for their increased adoption in the renewable energy market.

#### 3.3.3 Other Methods

Beyond traditional solution processing methods, alternative fabrication techniques for transport layers in OSCs are gaining prominence, driven by the need for improved efficiency, scalability, and material versatility. Among these, thermal evaporation [105, 106], pulsed laser deposition (PLD) [107], and magnetron sputtering [108] were reported to fabricate transporting layers in OSCs.

Thermal evaporation allows for the deposition of organic and inorganic materials under vacuum conditions, resulting in films with excellent uniformity and reduced impurities. This technique has been effectively utilized for various transition metal oxide interlayer materials, such as MoO_3_, NiO, V_2_O_5_, and WO_3_, which exhibit high work functions and good electrical conductivity. Yang et al. thermally deposited MoO_3_ onto ITO films using a conventional vacuum evaporation method, allowing precise control over the MoO_3_ layer thickness (5 nm), which resulted in an optimal PCE of 3.3% [105]. Yoo et al. discovered that the uniform amorphous WO_3_ film, deposited by thermal evaporation on ITO, displayed relatively low surface energy [106].

NiO, functioning as a p‐type oxide semiconductor, was deposited via PLD directly onto cleaned ITO as an interfacial layer for P3HT:PCBM‐based OSCs, achieving a remarkable efficiency of 5.2% with a high *V*_OC_ of 638 mV and an FF of 69% [107]. This performance was attributed to the effective hole‐transport and electron‐blocking properties of the 10 nm NiO layer. The deposition conditions, including O_2_ pressure and substrate temperature, significantly influenced the electrical and contact properties of transporting layers. Among these, O_2_ plasma treatment could enhance the electronic properties of the transporting film, yielding a more favorable work function for OSCs. The annealing temperature was critical for forming high‐performance transporting layers in OSCs. Additionally, NiOx thin films with a thickness of 3 nm, prepared by RF magnetron sputtering using a NiO target as the HTL in conventional OSCs with (PCPDTBT):PC71BM, exhibited superior performance compared to PEDOT:PSS and even thermally evaporated MoO_3_ [108].

The exploration of alternative processing methods for fabricating transport layers in OSCs is essential for advancing the technology and improving device performance. Optimization strategies such as doping, surface modification, and compositing are employed to address the inherent low conductivity and enhance the performance of the interlayer. Continued research in these areas will be vital for enhancing the efficiency and commercial potential of OSCs.

## 4 The Development of Electrodes

### 4.1 The Development of Anodes

The evolution of electrodes in OSCs reflects the ongoing progress in materials science and photovoltaic conversion technology. For anodes in conventional OSCs, it is essential to achieve an appropriate work function, as well as high transparency and conductivity. Early in 1986, the first OSC device with a planar heterojunction used ITO as the anode [109]. ITO, as the transparent conductive oxide, has good transparency (>80%) and low sheet resistance (>20 Ω sq^−1^), making it the standard electrode for OSCs.

However, ITO has several drawbacks, including limited sources, poor transparency in the blue region, intrinsic mechanical brittleness, and an energy‐intensive process [110, 111]. Therefore, it is imperative to investigate novel anode materials that can address the limitations of ITO. In the early twentieth century, anode materials including conductive polymeric layers [112], carbon nanotubes [113], other conductive oxides [114], metal grids [115], and metal nanowires [116] were developed. The development of the anode in OSCs has progressed from a single material of ITO to a diversified material system. Each step of evolution aims to overcome the limitations of the previous generation of materials and achieve higher conductivity, lower manufacturing costs, and greater practicality.

In contemporary research, despite the development of various anode materials, ITO continues to be the predominant choice for the anode in OSCs [117]. In the future, as materials science and nanotechnology continue to advance, it is expected that more innovative materials will be applied to the anode of OSCs, driving the continuous development of the entire industry.

### 4.2 The Development of Cathodes

Similar to anodes, cathodes in OSCs also necessitate good conductivity and an appropriate work function. In the late 19th century, specifically during the 1880s and 1890s, the cathode materials used in OSCs were predominantly metallic electrodes such as In, Al, Ca, Cu, Ag, among others [118]. Around the year 2000, the bilayer LiF/Metal cathodes were introduced [119]. Compared with metallic electrodes, LiF/Metal electrodes offer advantages such as a more suitable work function and enhanced stability [120]. Subsequently, the LiF layer was replaced with other superior buffer layers [121]. In contemporary research, metallic electrodes such as Ag and Al have once again emerged as the primary choice for conventional OSCs. This resurgence is attributed to advancements in buffer layers, including PDINN and PDINO [122].

Over the past decade, functionalized OSCs have garnered significant interest from researchers. For functionalized OSCs such as semitransparent or flexible OSCs, it is necessary for the cathode to possess high transparency or good flexibility. Numerous materials have been developed including ultrathin metals [123], metal nanowires [124], metal oxides [125], carbon‐based materials [126], and conductive polymer electrodes [127]. Each material possesses distinct benefits and drawbacks. Looking ahead, ongoing research will continue to refine existing materials and explore new compositions to meet the dual demands of transparency and flexibility more effectively.

### 4.3 The Processing of Electrodes

#### 4.3.1 Thermal Evaporation

OSCs have achieved remarkable advancements over the past few decades, with thermally evaporated electrodes emerging as the preferred top electrodes for fabricating high‐performance opaque OSCs. Depositing the electrode material by thermal evaporation is a common process used in OSCs. Thermal evaporation offers several advantages, such as precise control over film thickness, the ability to produce high‐purity films, and compatibility with a wide range of materials. Despite its disadvantages, including high energy consumption, strict requirements on vacuum conditions, and possible thermal damage of interface, thermally evaporated electrodes still predominate opaque OSCs.

**FIGURE 5:**
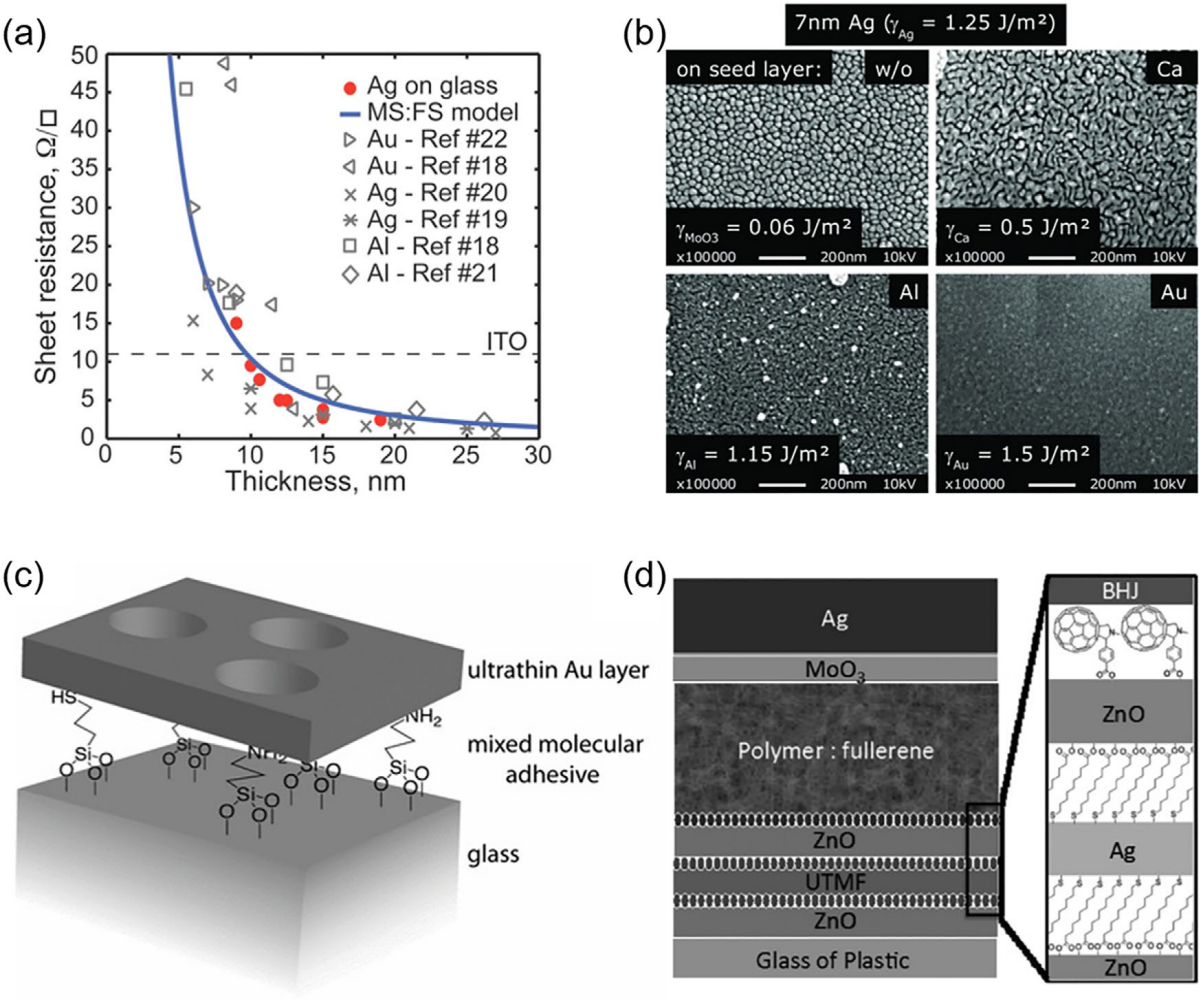
(a) A plot of experimentally determined sheet resistance vs metal film thickness. Reproduced with permission from Ref. [123]. Copyright 2008, AIP Publishing. (b) Scanning electron micrographs of 7 nm thick silver layers deposited on various seed layers with different surface energies γ taken from literature. Reproduced with permission from Ref. [134]. Copyright 2013, Wiley‐VCH. (c) A schematic illustration of an ultrathin Au film supported on a mixed MPTMS:APTMS monolayer on glass. Reproduced with permission from Ref. [135]. Copyright 2011, Wiley‐VCH. (d) OSC devices and the molecular structure of MUA and C60‐SAM employed for interfacial modifications. Reproduced with permission from Ref. [137]. Copyright 2014, Wiley‐VCH.

Except for application in opaque devices, thermally evaporated electrodes are utilized in semitransparent OSCs (ST‐OSCs) as well. Thermally evaporated electrodes are primarily ultrathin metals, among which ultrathin Ag electrodes are most widely applied. Unlike opaque devices, electrodes in ST‐OSCs are deposited with a thinner layer to enhance transparency. However, Shtein et al. proposed that a reduced thickness of the Ag layer led to an increase in sheet resistance (Figure 5a) [123]. With respect to this situation, Bernède et al. pointed out that 10 nm was an appropriate thickness for Ag when deposited at a rate of 0.20 nm/s [128]. The precise control of electrodes’ thickness is of great significance for ST‐OSCs using ultrathin metal electrodes [129, 130]. Our group investigated the tradeoff between conductivity and transparency of ultrathin Ag top electrodes, aiming to maximize the superiority of the novel small‐bandgap electron acceptor ATT‐2 [129]. When the thickness of electrodes increased from 5 nm to 20 nm, an enhancement in *J*_SC_ (from 13.38 to 18.53 mA cm^−2^) and a reduction in transparency (from 48% to 37%) were observed.

The increased resistance observed from a few nanometers of Ag electrodes was contributed to the formation of isolated nuclei, which caused aggregation during deposition [131]. Aggregation can be mitigated through methods such as introducing a molecular surfactant [132], constructing a DMD (dielectric/metal/dielectric) structure [133], or depositing a seed layer [134]. These methods can be effectively utilized as the top electrodes in OSCs. Molecular surfactants not only effectively modify the energy level alignment at the organic/metal cathode interface but also enhance electron extraction and photocurrent generation. Jen et al. employed an ultrathin fullerene‐containing surfactant to optimize the ST‐OSC devices, yielding a PCE of 4.22% and an AVT of 31.71%. DMD electrodes exhibit outstanding thermal, mechanical, and environmental stabilities, together with opto‐electrical properties [132]. Cho's group investigated the impact of metal thickness within the DMD structure, specifically concerning the inevitable loss of incident light attributed to the presence of metal [133]. Based on the modified MoO_3_/Ag/MoO_3_ electrodes, the ST‐OSC devices attained a PCE of 8.4% with an AVT of 25%. Seed layers demonstrate significant potential in promoting the formation of a continuous, smooth ultrathin metal film in the DMD structure. Leo et al. proposed that an ultrathin seed layer could be introduced between the metal layer and the underlying dielectric layer in a DMD structure to adjust the surface energy between these layers [134]. Several materials for the seed layer, including Ca, Au and Al were evaluated (Figure 5b). The results indicated that the optimal performance was achieved with a combination of 7 nm Ag and 1 nm Au, yielding a transmittance of 83% (at 580 nm) and a square resistance of only 19 Ω sq^− 1^.

As for bottom electrodes, ITO is the most commonly used material. ITO electrodes, nevertheless, do exist a few drawbacks mentioned above. This has prompted researchers to actively explore alternative bottom electrode materials. When molecular surfactants are introduced onto the substrate, several advantages can be achieved, including realigned interfacial energy levels, enhanced charge collection, and improved interfacial exciton dissociation. Hatton's group employed two molecular surfactants, namely APTMS (3‐aminopropyltrimethoxysilane) and MPTMS (3‐mercaptopropyltrimethoxysilane), to fabricate ultrathin transparent Au bottom electrodes (Figure 5c) [135]. Thiol functionalized silanes were investigated to interact strongly with both the substrate and the incoming metal atoms. The deposited 8.4 nm Au electrodes exhibited a low sheet resistance (12.2 Ω sq^−1^) and a low root‐mean‐square (RMS) roughness (about 0.4 nm). Compared with OSC devices of the same structure employing an ITO glass electrode, the device based on Au electrode exhibited a comparable *V*_OC_ and FFs, with only a slight decrease in *J*_SC_ (from 11.0 to 9.9 mA cm^−2^). Zhang et al. developed a bottom electrode featuring a DMD structure composed of PVK/Ag/(PVK or PEDOT:PSS), yielding a sheet resistance below 10 Ω sq^− 1^ and a maximum transmittance over 85% [136]. The OSC devices utilizing this electrode achieved a PCE of 2.95%, lower than their ITO counterparts. Jen's group utilized both surfactants and a DMD structure (Figure 5d), yielding an ultrathin Ag film with low RMS roughness of 0.95 nm and improved sheet resistance of 8.61 Ω sq^−1^ [137]. Between the ZnO layer (the dielectric layer) and Ag film (the metal layer), a dipolar SAM (self‐assembled monolayer) MUA was introduced to realign the interfacial energy levels, thereby achieving Ohmic contact and enhancing charge collection. Additionally, a fullerene‐based SAM (C_60_‐SAM) was applied on top of the ZnO layer to passivate the inorganic surface traps and improve interfacial exciton dissociation between ZnO and the active layer. Based on these two strategies, the OSC devices attained a PCE of 6.59% and 6.04% when fabricated on glass and plastic substrates, respectively.

#### 4.3.2 Solution Processing

Solution processing is another widely utilized method for the fabrication of electrodes. This technique offers numerous advantages, including ease of fabrication and lower energy consumption compared to vacuum deposition methods. Additionally, it is well‐suited for roll‐to‐roll manufacturing and large‐scale production. Next, we will categorize the electrodes into top and bottom sections, subsequently further classifying each segment by different material types to incorporate solution‐processed electrodes.

**FIGURE 6:**
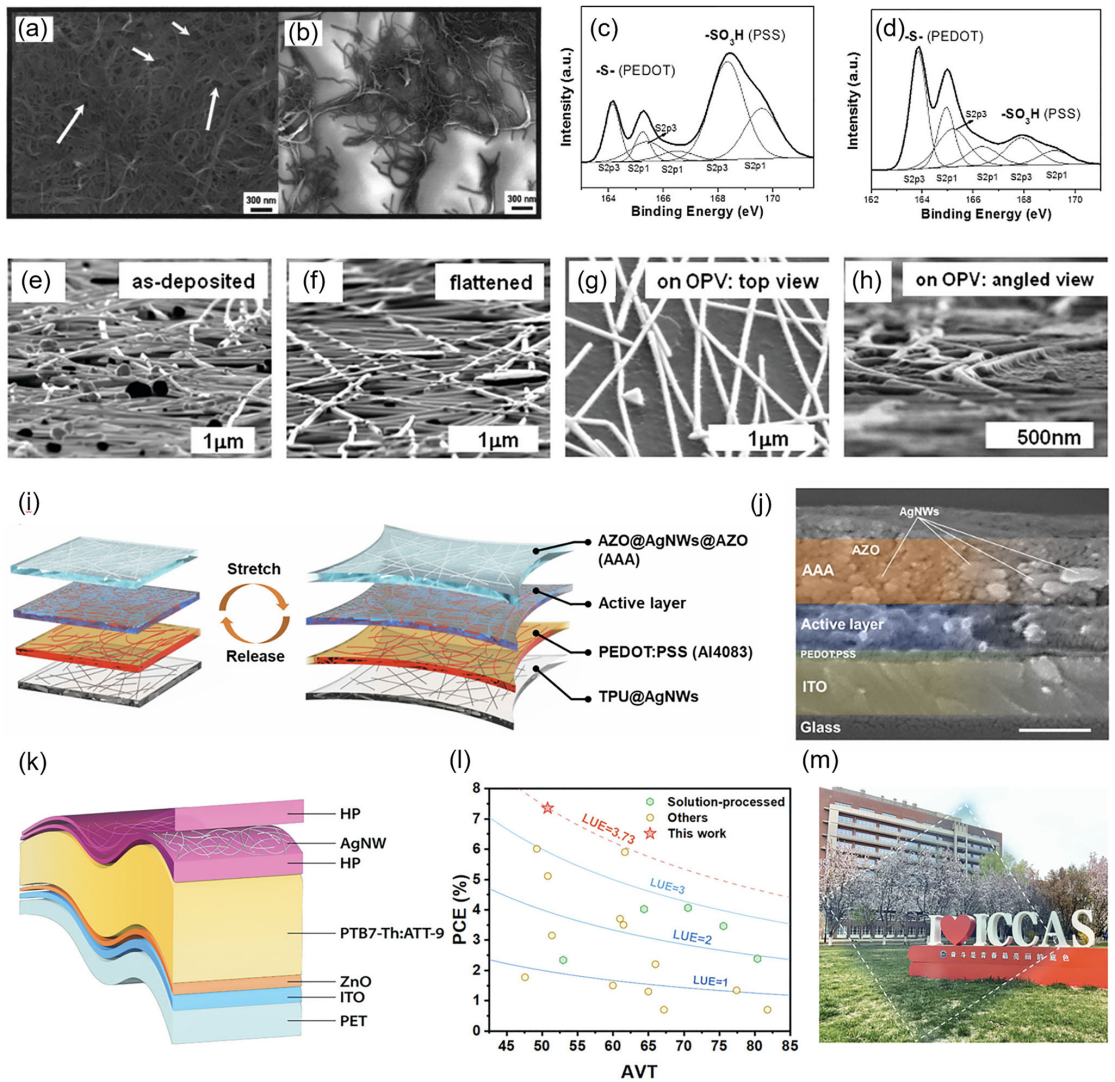
(a) SEM images of SWNT films deposited on glass, from 50 mL SWNT solution (b) and 10 mL SWNT solution. Reproduced with permission from Ref. [113]. Copyright 2005, AIP Publishing. Fitted S 2p XPS spectra of (c) the pristine films and (d) the acid‐treated films. Reproduced with permission from Ref. [143]. Copyright 2018, Wiley‐VCH. Ag nanowires as‐deposited on a glass substrate recorded 85° off‐normal (e) before and (f) after application of 104 psi uniaxial pressure was applied. Complete ST‐OSC cell seen from (g) the normal direction, and (h) 85° off‐normal. Reproduced with permission from Ref. [144]. Copyright 2010, American Chemical Society. (i) Schematic diagram of the ST‐OSC based on the configuration of TPU@AgNWs/PEDOT:PSS/active layer/AAA (j) and cross‐sectional SEM image of the OPV device. Reproduced with permission from Ref. [145]. Copyright 2023, Royal Society of Chemistry. (k) Illustration of a flexible ST‐OSC with sandwich‐structure transparent electrode, (l) statistics of reported PCE and AVT of high transmittance (>45%) ST‐OSCs without optical modulation, and (m) photograph of a high transmittance ST‐OSC with solution‐processed 5 mg mL^−1^ AgNW as electrode. Reproduced with permission from Ref. [124]. Copyright 2023, Wiley‐VCH.

Materials for bottom solution processed electrodes are available in a variety of options, including carbon‐based electrodes [138], metal nanowire electrodes [139], and conductive polymer electrodes [140]. Carbon nanotubes (CNT) are 1D carbon‐based materials with high conductivity and high transparency. Chhowalla et al. utilized single‐wall carbon nanotube (SWCNT) to function as the bottom electrode replacement of ITO [113]. The thickness of the SWCNT electrodes was modulated by varying the volume of the SWCNT suspension (Figure 6a,b). The highest PCE (1%) was achieved with the electrode thickness of 300 nm, which facilitated a 3D connection with the polymeric active layer. Graphene has gained significant research attention owing to its remarkable properties, including exceptional carrier mobility, high transparency in both visible and near‐infrared light, and considerable flexibility. Zhang's group developed reduced graphene oxide (rGO) films to serve as the bottom electrodes in OSCs [138]. The thickness of rGO films was meticulously controlled through spin‐coating conditions followed by the application of a polymethyl methacrylate (PMMA) film to provide structural support. The PMMA/rGO film was subsequently transferred onto polyethylene terephthalate (PET) substrates, functioning as transparent and conductive electrodes for OSC devices. A trade‐off between transparency and sheet resistance was observed, with the lowest achieved sheet resistance being 720 Ω sq^− 1^ for an rGO thickness of 28 nm thick rGO, which exhibited a transmittance of approximately 40%. Notably, the highest PCE recorded was 0.77% with an optimal rGO thickness of 21 nm. A significant advantage of AgNWs lies in the light‐scattering properties exhibited by NW networks, in addition to their high transparency and conductivity. Oh et al. deposited AgNWs onto the PET substrates using an acrylic resin buffer layer through a bar coating process [141]. After annealing, the AgNWs were effectively embedded within the acrylic resin. The presence of the acrylic resin buffer layer resulted in an RMS roughness of 18.4 nm for the AgNWs electrodes, significantly lower than the value of 97.8 nm observed without the buffer layer. Furthermore, this acrylic resin buffer layer markedly enhances the adhesion of AgNWs. With improved light scattering and trapping effects from the AgNWs electrodes, the OSC devices achieved a peak PCE of 7.58%. Son's group developed ultrasmooth MXene‐AgNWs hybrid electrodes through a layer‐by‐layer process [142]. The MXene/AgNW/colorless polyimide (cPI) hybrid electrode was fabricated using a reverse sequential processing method involving MXene nanosheets and AgNWs, resulting in significantly low sheet resistance (13.08 Ω sq^−1^) and high transmittance (79% at 550 nm). OSC devices based on the hybrid electrodes achieved a PCE of 10.3%. PEDOT:PSS exhibits several characteristics, including high transmittance in the visible spectrum, exceptional thermal stability, adjustable electrical conductivity, as well as good film‐forming capability achieved through various fabrication techniques. Bao et al. employed a fluorosurfactant for PEDOT:PSS flexible transparent electrodes [140]. The incorporation of the fluorosurfactant resulted in PEDOT:PSS films demonstrating an enhanced sheet resistance (46 Ω sq^−1^ at 82% transparency), along with improved compatibility on hydrophobic surfaces. When these films were deposited onto a pre‐strained PDMS substrate, no changes in sheet resistance were observed over more than 5000 cycles of strain ranging from 0% to 10%. 4‐layer PEDOT: PSS‐based OSC devices reached a PCE of 2.22%. Ge's group introduced a novel gentle acid treatment conducted at room temperature, which enhanced the performance of PEDOT:PSS bottom electrodes [143]. When treated with methanesulfonic acid (CH_4_SO_3_), PEDOT:PSS films demonstrated an RMS of 2.14 nm. A rearrangement of PEDOT:PSS films involving the removal of PSS has been achieved, resulting in an enhancement of film conductivity (Figure 6c,d). This improved morphology and conductivity facilitate effective interconnection among conducting PEDOT chains, thereby promoting charge hopping and resulting in a significant reduction in sheet resistance to 2.9 Ω sq^−1^. The all‐solution‐processed OSC devices showed an impressive PCE over 10%.

Materials for top solution‐processed electrodes are mainly metal nanowire electrodes and conductive polymer electrodes [144, 145, 146]. Nanowire electrodes offer excellent mechanical and photoelectric properties, as well as solution‐processability. Among these, Ag nanowires (AgNWs) are preferred due to their low square resistance, high transmittance, and good flexibility. Peumans et al. developed a laminated AgNWs as top electrodes (Figure 6e–h) [144]. The process could be roughly divided into three steps. First, the nanowire suspension was dropped onto a cleaned glass substrate and dried for 10 min. Then, a razor blade was used to pattern the AgNWs into electrode shapes. Last, the AgNWs were laminated onto the OSC structure under a certain pressure. The PCE achieved was 57% of that of a device with a conventional metal cathode. Unlike bottom electrodes, common post‐treatments for AgNWs, such as high‐temperature annealing, plasma, and reducing agents, are unsuitable for top electrodes as they will inevitably damage the active layer. Advancing the processing of transparent top electrodes is of great significance for the development of ST‐OSCs. Li's group developed a full‐solution‐processed structure for transparent top electrodes, AZO@AgNWs@AZO (Al‐doped zinc oxide) (Figure 6i,j) [145]. With reinforced interfacial stability and an offered 3D long‐range pathway for efficient charge transport and collection, the ST‐OSC device obtained 12.83% PCE with 26.7% AVT. Thermoplastic elastomer (TPU) is utilized as the substrate of the front electrode (AgNWs) for addressing the mechanical stability of flexible electrodes. The TPU‐embedded AgNW electrodes exhibited exceptional durability, maintaining over 91% conductivity at a bending radius as small as 0.1 mm and showing less than 2% variation in resistance after 5000 bending cycles. Furthermore, these electrodes sustained functionality under 100% tensile strain and minimal conductivity degradation over 4300 stretch‐release cycles, highlighting their potential for mechanically robust flexible solar cells. Zhu's group introduced a sandwich structure HP/AgNWs/HP, which improves the wettability and conducting properties of AgNWs. HP comprised HMO:PEDOT:PSS:Triton (Figure 6k–m) [124]. HMO served as a hole transporting material compatible with AgNWs. The addition of PEDOT:PSS slightly enhanced the energy level for better alignment with the donor's HOMO level. Triton was included to improve ink processability. ST‐OSC devices with these electrodes reached a PCE of 7.34% with a high AVT over 50%. Zhou et al. embed large‐area silver nanowire electrodes into polymer substrates for reduced surface roughness and suppressed electrical shunt, significantly boosting the photovoltaic efficiency and illumination stability of the modules, with large‐area flexible modules achieving high certified efficiencies and retaining a substantial portion of their initial efficiency after prolonged continuous illumination [147]. Solution‐processed conductive polymer top electrodes (mainly PEDOT:PSS) demonstrate several advantages, including rapid processing, energy efficiency, cost‐effectiveness, and transparency [148]. For PEDOT:PSS top electrodes, there are challenges associated with commercially available high‐conductivity PEDOT:PSS formulations. These formulations exhibit poor wetting properties on hydrophobic active layers, and the thinness of the active layer makes it susceptible to penetration [149]. By mixing two types of PEDOT:PSS (PH‐1000 and CPP 105D), Kippelen's group developed a PEDOT:PSS blend that exhibited excellent wetting properties on the active layer [150]. This advancement in OSC devices resulted in a PCE of 2.4%. Inspired by a traditional Chinese calligraphy technique, Zhou's group developed an innovative solution‐processed method for fabricating organic solar cells and modules utilizing the “Maobi” tool [151]. With the assistance of Maobi coating, the thickness of conducting polymer electrode films could be precisely tuned and controlled, ranging from several tens of nanometers to one micrometer. The 18 cm^2^ modules exhibited a PCE of 6.3%.

#### 4.3.3 Other Methods

Other process methods for electrodes in OSCs include sputtering, chemical vapor deposition (CVD), and printing technologies. Sputtering is a process similar to thermal evaporation, capable of producing doped metal films. CVD primarily serves to synthesize carbon‐based materials, such as graphene and carbon nanotubes. Printing technologies encompass a wide range of aspects and materials; in this discussion, we will specifically focus on the printing of metal grids.

**FIGURE 7:**
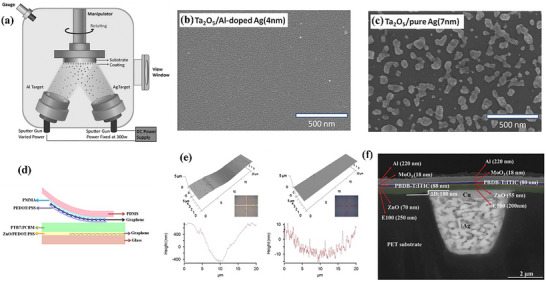
(a) Set‐up of co‐deposition of Ag and Al. Reproduced with permission from Ref. [152]. Copyright 2014, Wiley‐VCH. (b) SEM images of Ta2O5/Al‐doped Ag (4 nm). (c) SEM images of Ta_2_O_5_/ pure Ag (7 nm). Reproduced with permission from Ref. [153]. Copyright 2015, Wiley‐VCH. (d) Device structure of ST‐OSCs with all‐graphene electrodes. Reproduced with permission from Ref. [126]. Copyright 2015, American Chemical Society. (e) AFM and optical microscope image of a pattern filled with nano‐silver paste and heat treated and a pattern paste filled followed by coating with PEDOT:PSS. Reproduced with permission from Ref. [156]. Copyright 2013, Elsevier. (f) Cross‐section SEM images of the flexible PBDB‐T:ITIC device. Reproduced with permission from Ref. [158]. Copyright 2019, Wiley‐VCH.

Sputtering is a widely employed deposition technique in the fabrication of various thin films, including industrial ITO electrodes. This method facilitates the production of high‐quality, crystalline, and uniform films; however, it necessitates conditions of high vacuum and elevated temperatures. Through the sputtering process, Guo's group developed Al‐doped Ag films (Figure 7a), which were utilized as bottom electrodes in OSC devices [152]. The incorporation of Al effectively suppresses the 3D island growth of Ag and facilitates the formation of ultrathin films, eliminating the need for seeding layers and critical fabrication conditions. With a low RMS value of 0.78 nm, the 7 nm thick Al‐doped Ag electrodes facilitated the achievement of PCE of 7.44%, surpassing that of the ITO‐based device. Then, they introduced T_2_O_5_ beneath the Al‐doped Ag film to serve as a wetting layer and an optical cavity resonance (Figure 7b,c) [153]. With the incorporation of T_2_O_5_, the thickness of the Al‐doped Ag film was reduced to 4 nm. By controlling the thickness of T_2_O_5_, the PCE of 8.57% was achieved based using a Ta_2_O_5_/Al‐doped Ag electrode.

CVD is considered the most effective method for synthesizing graphene thin films, as it enables the production of large‐area, uniform, and high‐quality graphene, though this method requires high temperature for the decomposition of carbon precursor. Yan et al. developed all‐graphene electrodes for ST‐OSCs (Figure 7d) [126]. These graphene electrodes were fabricated through the CVD process and subsequently transferred and stacked onto substrates. For the graphene anode, a two‐layer stacked graphene film was coated with a thin layer of PEDOT:PSS, resulting in a reduced sheet resistance of approximately 170 Ω sq^− 1^, compared to the uncoated film's sheet resistance of 320 Ω sq^−1^. In the case of the graphene cathode, incorporating ZnO into the PEDOT:PSS/graphene film facilitated energy level adjustment, yielding a sheet resistance of 230 Ω sq^− 1^. The ST‐OSCs utilizing all‐graphene electrodes achieved a PCE of 3.4% with an AVT of 40%. Zhou's group introduced a low‐temperature CVD process to fabricate graphene electrodes, achieving an average sheet resistance of 294 Ω sq^−1^ and optical transparency of 65% at a wavelength of 500 nm [154]. The graphene electrodes were synthesized at a growth temperature of 600°C using chlorobenzene encapsulated in poly(methyl methacrylate) (PMMA) polymer on copper (Cu) foil substrates. A 5 mm^2^ OSC device utilizing these electrodes attained a PCE of 1.27%. Leo et al. investigated free‐standing multi‐wall carbon nanotube (f‐CNT) sheets as top electrodes [155]. Initially produced through a CVD process, the f‐CNT sheets were subsequently immersed in orthogonal liquid hydrofluoroether for several seconds to enhance their density, resulting in a sheet resistance of 250 Ω sq^−1^. The fabricated OSC devices achieved a PCE of up to 1.5%.

Printing technologies offer significant advantages for patterning and the fabrication of large‐area devices on a roll‐to‐roll basis, particularly in the context of regular metal grid structures. Kim's group embedded Ag grids into a polyethylene naphthalate (PEN) film to serve as the bottom electrodes [156]. Through the doctor blade method, nano‐silver paste was filled into the imprinted micro‐scale channel patterns (Figure 7e). The resulting Ag grid electrodes demonstrated a low sheet resistance of 6.85 Ω sq^−1^ and an impressive transparency of 80.35%, contributing to a PCE of 1.40% in OSC devices. Aiming to incorporate metals into polymers as well, Kang et al. initially printed Ag grids through a gravure printing method prior to applying the polyimide (PI) solution [157]. Following a thermal curing process, the Ag‐grid‐embedding flexible substrate was detached from the glass substrate. Subsequently, a 20 nm layer of amorphous ITO was deposited onto the flexible substrate via a sputtering process to fabricate transparent conducting electrodes. The highly flexible transparent conducting electrodes achieved an impressive transparency of 93% at 550 nm, a low sheet resistance of 13 Ω sq^−1^, and remarkable flexibility with a bending radius of 200 µm. These attributes contributed to the highest PCE of 4.01% in OSC devices. Ma's group developed a flexible Ag/Cu composite grid electrode exhibiting outstanding low sheet resistance (less than 1 Ω sq^−1^) [158]. The composite electrode features high‐resolution hexagonal silver and copper grids, which measure 3 µm on PET substrates (Figure 7f). Notably, the majority of the area remains unoccupied, resulting in a high transparency of 84%. Utilizing this electrode, a large‐area (1 cm^2^) flexible OSC device achieved a PCE of 12.26%.

## 5 The Development of Large‐Area Devices

In the quest for the successful commercialization of OSCs, the development of scalable large‐area manufacturing technologies is of paramount importance [159]. The transition from laboratory‐scale devices to industrial‐scale production necessitates techniques that can maintain performance while accommodating the increased area of the substrates. This review delves into the advancements and challenges associated with large‐area device fabrication in OSCs, focusing on meniscus coating, gravure printing, inkjet printing, and screen printing as key scalable technologies.

Blade coating, also known as doctor blade coating, has been recognized as a feasible meniscus coating technique for fabricating high‐quality large‐area photovoltaic devices [160]. Blade coating involves the application of a thin layer of ink onto a substrate by a blade, with the printing quality and film thickness being controlled by processing speed, the surface energy of the substrate, and the gap between the blade and the substrate. However, blade coating faces challenges in adapting to high‐throughput printing technologies due to discontinuous ink supply. Other coating techniques, such as slot‐die coating, gravure printing, inkjet printing, and screen printing, are applicable to high‐throughput printing technology, although they require more sophisticated equipment [161, 162]. Among these, slot die coating is another meniscus coating technique that controls layer thickness by adjusting the pumping rate, printing speed, and width of the printed area. Gravure printing stands out for its high throughput rate and precision, which involves the transfer of ink from a gravure cylinder to the substrate, with the ink being picked up by the engraved cavities on the cylinder and then transferred to the substrate with high resolution. Inkjet printing is a digitally controlled, contactless patterning technique that offers the advantage of fine control over droplet size and trajectory, leading to high‐resolution and reproducible digital patterns with minimal material loss. It is compatible with roll‐to‐roll manufacturing, which is crucial for the commercialization of OSCs. However, the slow printing speed and the need for low‐viscosity inks limit its application in large‐area OSCs. Screen printing is another method that can be integrated into roll‐to‐roll manufacturing processes. It involves the transfer of ink through a patterned mesh screen, which defines the thickness of the printed film. This method is limited by the need for high‐viscosity inks to avoid pattern deviation and low‐volatility solvents to prevent thickness variation due to evaporation‐induced concentration gradients.

**FIGURE 8:**
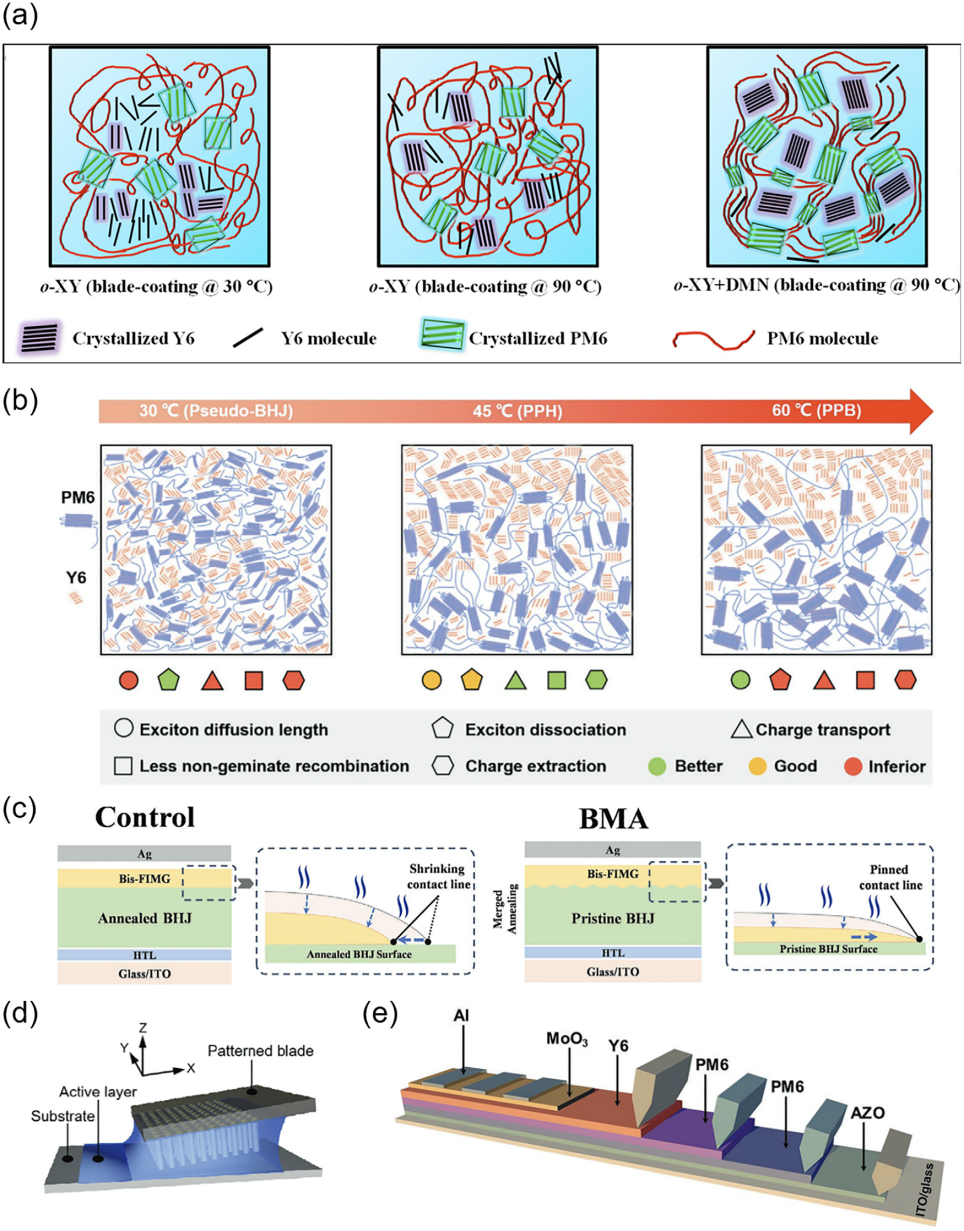
(a) Proposed morphological evolution for the PM6:Y6 blend films prepared with different conditions. Reproduced with permission from Ref. [165]. Copyright 2021, American Chemical Society. (b) The evolution of morphological characteristics and fundamental processes as a function of baseplate temperature. Reproduced with permission from Ref. [166]. Copyright 2021, Wiley‐VCH. (c) Schematic illustration of device structures and drying dynamics of the ETL meniscus via different processing. Reproduced with permission from Ref. [167]. Copyright 2022, Wiley‐VCH. (d) Schematic illustration of patterned blade coating. Reproduced with permission from Ref. [168]. Copyright 2021, Wiley‐VCH. (e) Schematic illustration of the RS‐LBL blade‐coating process for large‐area OSC modules. Reproduced with permission from Ref. [169]. Copyright 2022, Wiley‐VCH.

The primary limitation of these scalable methods lies in the challenge of controlling film uniformity, thickness, crystallization, and morphology. Following the scalable coating process, the ink solvent needs to evaporate in a regulated way to produce a stable, evenly distributed, and appropriately structured film. Therefore, ensuring printing quality with well‐formed crystallization and phase separation during film development is crucial. Printing parameters—such as solvent choice, additives, and coating temperature—often differ significantly from those used in laboratory‐scale device production. The active layer of most state‐of‐the‐art small‐area devices was normally processed by the low boiling point solvent, chloroform, which presents a narrow processing window. Zhou et al. investigated the relationship between acceptor structure and processing solvent for high‐performance and long processing windows in large‐area processing [163]. It is found that long side chains on the pyrrole rings of the acceptors enable favorable morphology and decent photovoltaic performance when processed by a high‐boiling‐point solvent (chlorobenzene) with a wide processing window. The large‐area module (over 25 cm^2^) via blade coating shows a PCE of 14.07%. The incompatibility of high‐boiling‐point solvents with high‐speed printing processes poses a critical challenge for the development of large‐area devices. Min et al. applied a co‐solvent approach (chloroform and chlorobenzene) to meticulously adjust the film formation process, thereby attaining an optimal BHJ structure [164]. Their work demonstrates that the co‐solvent tightly regulates the film formation, leading to a blend with high domain uniformity and an appropriate phase‐separated network. Consequently, a PCE of 16.17% was realized in the blade‐coated PM6:Y6‐2Cl system, surpassing any post‐treatment, and significantly outperforming devices processed with chloroform or chlorobenzene alone, which yielded 14.08% and 11.44%, respectively. Xie and colleagues incorporated the additive 1,2‐dimethylnaphthalene (DMN) into the PM6:Y6 blend to enhance the film crystallization (Figure 8a) [165]. Including 0.5% volume fraction DMN led to a notable blue shift in the Y6 absorption peaks, suggesting a reduction in Y6 aggregation. Moreover, the high‐boiling, non‐halogenated solvent DMN fosters additional nucleation points, thereby boosting the crystallinity of both PM6 and Y6. As a result, the OSCs, with an active area of 1.0 cm^2^, achieved an impressive PCE of 13.87%. The coating temperature is crucial for controlling the crystallization process. Min et al. thoroughly investigated how the substrate temperature affects the photovoltaic performance of devices based on PM6:Y6, utilizing sequential blade‐coating deposition technology [166]. They observed that as the temperature rises, the active layer's morphology experiences a clear transformation from a pseudo‐BHJ at 30°C to a pseudo‐planar heterojunction at 45°C, and then to a pseudo‐planar bilayer at 60°C (Figure 8b). With its superior vertical phase distribution, the pseudo‐planar heterojunction allows the blend film made at 45°C to have an optimal vertical composition gradient, leading to superior device performance compared to those prepared at lower or higher baseplate temperatures.

Significantly, various optimization strategies have been put forward to enhance the scalability of manufacturing processes. For instance, Li et al. introduced a guest‐assisted assembly approach to create a uniform large‐area device using blade coating [46]. This method optimizes the crystallization of the host material through intermolecular interactions. To prevent the formation of pinholes in the electron‐transporting layer, Chen et al. developed a bilayer merged annealing (BMA)‐assisted blade‐coating technique (Figure 8c) [167]. This technique effectively addresses the de‐wetting problems between polar charge transport layer solutions and non‐polar bulk heterojunction blends, thereby enhancing film coverage and the electronic and electric contacts of the multi‐stacked photoactive layers. Consequently, the device achieved a remarkable efficiency of 14.79% over an active area of 18.73 cm^2^. Regulating fluid dynamics is essential for the crystallization of molecules and the behavior of phase separation. Ma and colleagues crafted patterned blade micro‐cylinder arrays through photolithography to amplify both the extensional and shear strain rates of the fluids. Therefore, Ma et al. proposed a patterned blade coating strategy (Figure 8d) [168]. The combined impact of extensional and shear flow leads to a more compact packing and organization of PM6 polymer chains during the subsequent evaporation, while Y6 molecules achieve adequate crystallization. Moreover, the patterned blade facilitates the orderly orientation of molecules and enhances crystallinity, which in turn promotes an optimized phase separation. Consequently, the PM6:Y6 films coated with the patterned blade achieved a PCE of 15.93%, surpassing the 14.55% efficiency obtained from films coated with a standard blade. Li and colleagues have engineered a reversible and sequential layer‐by‐layer (RS‐LBL) deposition technique, which involves a double forward/reverse blade‐coating process for the polymer donor and a single forward blade‐coating for the Y6 acceptor (Figure 8e) [169]. This method allows for the precise manipulation of the fluid mechanics within the PM6:Y6 active layer. Their research revealed that the non‐Newtonian fluid properties of PM6 predominantly influence the wedge‐shaped mass distribution and the progressive reduction in film thickness along the blade‐coating direction within the active layer. Furthermore, the uneven phase aggregation and crystallization across a large active area can cause disparities in hole/electron mobility and increase charge recombination losses in the sub‐cells, adversely affecting the efficiency of solar modules. By employing the RS‐LBL approach, a uniform morphology with favorable phase separation and crystallization is achieved in an active layer spanning 10 × 10 cm^2^. Consequently, the RS‐LBL‐based OSCs demonstrate superior operational stability and an impressive PCE of 13.47% is realized, with significantly reduced charge recombination losses in a large‐area OSC module of 36 cm^2^.

While each of these scalable manufacturing technologies presents unique advantages and challenges, they are all critical in the development of large‐area OSCs. The choice of technology often depends on the specific requirements of the application, such as the desired throughput, resolution, and cost. To bridge lab‐scale innovations with commercial realities, it is essential to incorporate quantitative metrics from pilot‐scale production, including coating speeds (e.g., meters per minute in roll‐to‐roll processes), material utilization rates, and module‐level cost analyses ($/W). Beyond technical performance, reproducibility in large‐scale fabrication remains a persistent challenge, particularly in maintaining uniformity across meter‐scale substrates and ensuring consistent ink formulation stability over time. Furthermore, environmental considerations such as solvent recovery and the reduction of hazardous waste must be systematically addressed to align with sustainable manufacturing standards. Additionally, adopting standardized stability assessment protocols (e.g., ISOS series for light, thermal, and environmental stress) in both research and pilot‐scale studies will provide comparable and predictive lifetime data, accelerating technology transfer. Indeed, flexibility stands out as a key benefit of OSCs over conventional photovoltaic technologies, highlighting their unique commercial potential. To develop flexible, large‐scale devices, it is crucial to manufacture high‐quality flexible transparent electrodes that possess low electrical resistance, high optical transparency, a smooth surface, and excellent mechanical properties. Additionally, the large‐scale production of photoactive layers that are not sensitive to thickness and are cost‐effective is another critical requirement. These layers must maintain their performance regardless of variations in thickness, ensuring consistency and reliability in the final devices. As research progresses, it is expected that these methods will continue to evolve, addressing the current challenges and enabling the widespread commercialization of OSCs.

## 6 Conclusion

This review has provided a comprehensive analysis of the device fabrication processes of OSCs, emphasizing the critical roles of active layers, transport layers, and electrodes in determining overall device performance. We discussed the unique advantages of OSCs, including their lightweight and flexible nature, as well as the cost‐effective processing methods that facilitate their integration into diverse applications. The importance of optimizing the active layer morphology and enhancing charge transport through carefully designed transport layers has been highlighted as essential for improving power conversion efficiency. Additionally, the selection and processing of transporting layers and electrode materials play a significant role in charge extraction efficiency, necessitating ongoing research into alternative materials and deposition techniques.

Looking ahead, several key areas warrant further investigation. First, the development of new organic materials with improved light absorption and charge transport properties is crucial for enhancing device performance. Additionally, advancing scalable manufacturing techniques, such as roll‐to‐roll processing, will be vital for producing large‐area devices with consistent quality and efficiency. Research should also focus on enhancing the long‐term stability of OSCs under real‐world conditions, addressing issues related to moisture sensitivity and thermal degradation.

Finally, interdisciplinary collaborations that integrate materials science, engineering, and environmental studies will be essential in driving innovation in OSC technology. By addressing these challenges and focusing on the outlined research directions, the future of OSCs holds significant promise for contributing to sustainable energy solutions and meeting global energy demands. The ongoing advancements in this field not only enhance our understanding of OSC technology but also pave the way for its practical application in diverse sectors.

## Conflicts of Interest

The authors declare no conflict of interest.

## Data Availability

The authors have nothing to report.
